# A coming-of-age story: adult neurogenesis or adolescent neurogenesis in rodents?

**DOI:** 10.3389/fnins.2024.1383728

**Published:** 2024-03-05

**Authors:** Jon I. Arellano, Alvaro Duque, Pasko Rakic

**Affiliations:** ^1^Department of Neuroscience, Yale University, New Haven, CT, United States; ^2^Kavli Institute for Neuroscience at Yale, Yale University, New Haven, CT, United States

**Keywords:** adult neurogenesis, mouse, rat, adolescence, adulthood

## Abstract

It is surprising that after more than a century using rodents for scientific research, there are no clear, consensual, or consistent definitions for when a mouse or a rat becomes adult. Specifically, in the field of adult hippocampal neurogenesis, where this concept is central, there is a trend to consider that puberty marks the start of adulthood and is not uncommon to find 30-day-old mice being described as adults. However, as others discussed earlier, this implies an important bias in the perceived importance of this trait because functional studies are normally done at very young ages, when neurogenesis is at its peak, disregarding middle aged and old animals that exhibit very little generation of new neurons. In this feature article we elaborate on those issues and argue that research on the postnatal development of mice and rats in the last 3 decades allows to establish an adolescence period that marks the transition to adulthood, as occurs in other mammals. Adolescence in both rat and mice ends around postnatal day 60 and therefore this age can be considered the onset of adulthood in both species. Nonetheless, to account for inter-individual, inter-strain differences in maturation and for possible delays due to environmental and social conditions, 3 months of age might be a safer option to consider mice and rats *bona fide* adults, as suggested by The Jackson Labs.

## Introduction

Although we all have an intuitive idea of what adulthood is, it is not always an easy task to define when it starts (Rakic, 2002; Lindsey and Tropepe, 2006; Kempermann, 2011). Defining adulthood onset is especially difficult in species with rapid postnatal development such as murine rodents, the model of choice for most neuroscience studies. Indeed, a look at the research on inbred strains of rodents during the past century reveals a striking feature: there is no standard, consensual definition of adulthood or when it starts in rats or mice. Indeed, different authors and studies have different views on the subject (McCutcheon and Marinelli, 2009; Jackson et al., 2017). For example, long-term potentiation studies in rodent hippocampal slices are commonly performed on tissue from very young animals, frequently before puberty and even before weaning (McCutcheon and Marinelli, 2009).

Obviously, the definition of adulthood is particularly relevant for studies on adult neurogenesis, the generation of new neurons beyond the developmental period, that has been described in murine rodents in two regions: the subventricular zone (SVZ) of the lateral ventricle, that produces new neurons that will migrate to the olfactory bulb through the rostral migratory stream (Lim and Alvarez-Buylla, 2016) and the subgranular zone (SGZ) of the dentate gyrus of the hippocampus, that produces new granule cells that integrate in the dentate gyrus network (Kempermann, 2011). Given the functional relevance of the hippocampus, there has been much interest in unraveling the dynamics of the process in the dentate gyrus and its possible functional significance. For simplicity, we have focused this perspective on the field of adult hippocampal neurogenesis, although the points we raise apply also to analyses of adult neurogenesis in the olfactory system, and more generally to studies in murine rodents.

As mentioned above, it seems evident that the study of adult hippocampal neurogenesis (AHN) requires a well-accepted, consistent definition of adulthood, that has been, however, elusive. Different criteria have been cited: adulthood starts after weaning (at postnatal day 21), reasoning that by that age, the adult neurogenic niche, the SGZ, has acquired its definitive structure; adulthood starts with puberty, when sexual reproduction is achieved; and adult neurogenesis is mostly adolescent neurogenesis, assuming adolescence as part of adulthood (Kempermann, 2011). Beyond the ambiguity, there is a common denominator in those definitions to consider very young animals (post-weaned, puberal or adolescent) as adults. Indeed, if we would apply some of those criteria to our species, adulthood onset could be established either around 12 years old, the average age of puberty for females in the United States (Herman-Giddens, 2006), raising the question if a puberal 9-year-old female would be considered adult. Or alternatively, adulthood onset could be set at age 7, when the human SGZ exhibits its mature configuration (Sorrells et al., 2018), although younger ages have not been studied. Conversely, data from our own species suggest that adulthood is achieved at the end of the adolescent period, at about 19 years of age (Spear, 2013), and developmental processes such as myelination and synaptogenesis take decades to complete (Petanjek et al., 2011; Miller et al., 2012).

**Figure 1:**
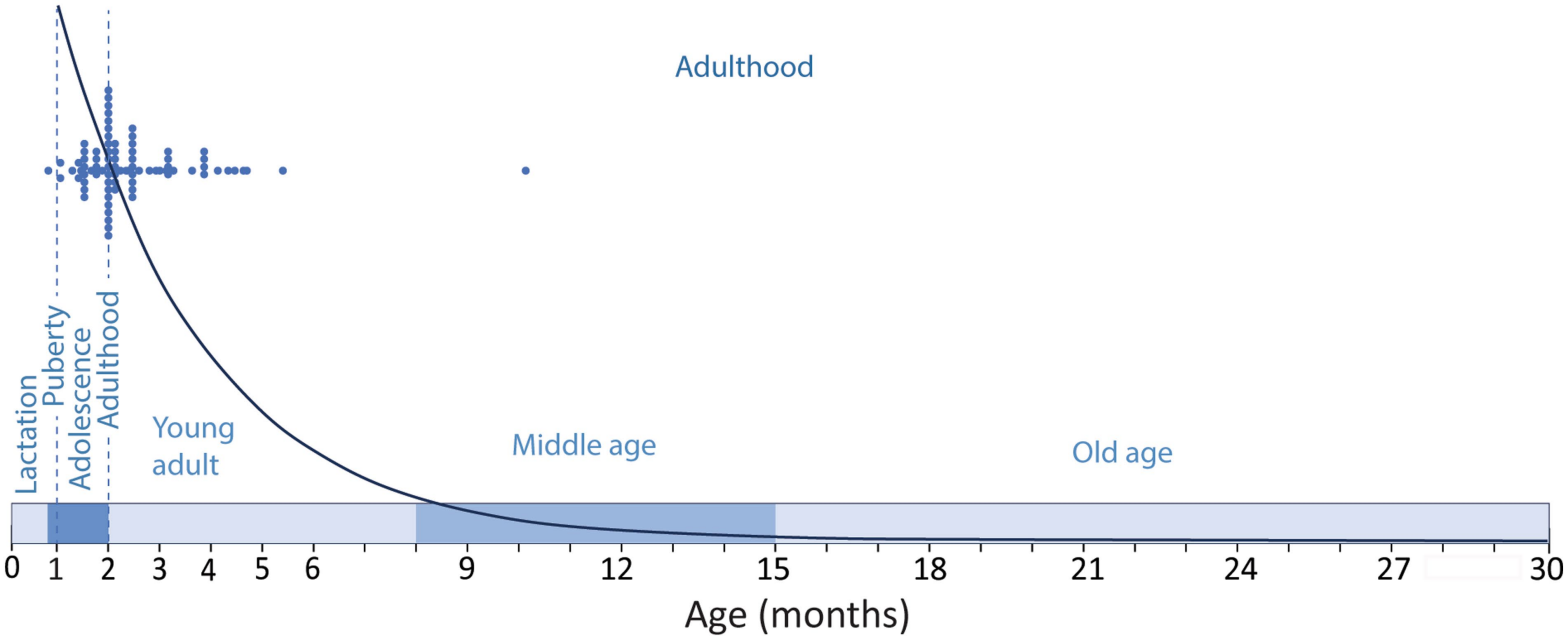
Timeline of the developmental stages along the lifespan of a C57 mice showing the period of lactation (until P21–P23); adolescence (~P23–P60); puberty (~P26–P30) and adulthood (~P60–P900). Blue dots show the ages of the experimental animals used in a random sample of functional studies on adult neurogenesis as described by Snyder (2019) compared to the modeled distribution of neurogenesis [y = 33110e^−0.39x^; regression of the number of DCX labeled cells in the mouse dentate gyrus across age (Ben Abdallah et al., 2010)]. Note that the selected studies are performed mostly in adolescent animals or in 2-5-month-old young adults, and only one study used middle aged animals, disregarding most of the adulthood period when neurogenesis is much lower.

The ambiguities in the definition of adulthood in rodents, and the bias toward very young animals are frequent in experimental research on adult neurogenesis, and different labs seem to have their own criteria to define adulthood. While many studies use 8-weeks-old or older animals, that can be considered adult (see below), it is not uncommon to find studies define 1-month-old mice as adults and probably the most common definition of adult lab rodents might be “6–8 weeks old animals” that, as reasoned below, would be adolescent, not adult (Flurkey et al., 2007; McCutcheon and Marinelli, 2009; Brust et al., 2015). In this regard, Snyder (2019) performed a meta-analysis of a random selection of 85 functional studies on adult neurogenesis from 1994 to 2018, and showed that 49% of the studies used animals younger than 60 days corresponding to juvenile or adolescent animals (Flurkey et al., 2007; McCutcheon and Marinelli, 2009; Brust et al., 2015), while only 7% used animals older than 4 months, and only 1% used animals older than 6 months (Figure 1). This last age, 6 months, is a better match for middle age rodents, although our review suggest middle age in C57 mice might extend from 8 to 15 months of age (see below), in agreement with data from The Jackson Labs defining middle age C57 mice as 10–14 months old (Flurkey et al., 2007). The general trend to consider very young animals as adults is striking, since in mammalian species, adulthood comprises most of the life of the individual (Figure 1). Therefore, it does not seem fair to use very young animals or to “force” the onset of adulthood to very young ages, running the risk of trespassing into developmental stages, while most of the adulthood period extending into middle-and old age is essentially disregarded. It would be more prudent (and scientifically sound) to choose an age when we are reasonably sure the animal is adult, beyond interindividual or inter-strain variability, moving the onset of adulthood toward an older age. In this regard, The Jackson Laboratories, an institution with a trajectory that supports an authoritative view on mice biology and reproduction, define adult mice as those aged 3-month-old or older (Flurkey et al., 2007), a reasonable threshold that would overcome the developmental differences mentioned above. Evidently, there are logistic and economic reasons in the current research climate that reward the use of young animals, mostly related to animal colony costs and shortening time for the collection of experimental data and ultimately for publication (Jackson et al., 2017). However, maybe the most relevant reason is related to the precipitous decline of neurogenesis with age (Kuhn et al., 1996; Ben Abdallah et al., 2010; Amrein et al., 2011). This dramatic and continuous decline is likely behind the tendency to use adolescent animals and young adults, focusing on a period of the life of rodents when neurogenesis in the dentate gyrus is abundant, but disregarding that middle age (~6–8 to 15 months old) and aged rodents (older than 15-months) exhibit low levels of neurogenesis (Figure 1).

Thus, the conclusions of those studies might be biased in two fundamental ways. First, they rely on very prolific adult hippocampal neurogenesis that might produce enough new neurons to make a functional difference, ignoring the subsequent decline in neurogenesis that reduces it to levels that might significantly lessen their functional relevance. And second, since some of the animals are not adult, their physiology and behavior might not be representative of the adult conditions. McCutcheon and Marinelli (2009) illustrated this last point with an eye-opening example: imagine a clinical trial for a novel analgesic or antipsychotic drug for adult patients performed in children. Obviously, it would be immediately disqualified, but that is a common occurrence in rodent research, and particularly in the field of adult neurogenesis. Indeed, there is a clear age mismatch between adult neurogenesis studies in rodents and in humans that might explain in part the controversy regarding the existence of adult neurogenesis in our species (Snyder, 2019). In the following section we will present a summary of the abundant literature about what defines adulthood and how that applies to rodents.

## Adulthood is better defined by sexual maturity than by sexual capacity

Traditionally, adulthood has been related to sexual reproduction, and the onset of adulthood has been typically described as the time when the animal reaches sexual maturity. This definition has been used liberally, equating sexual maturity with sexual capacity, and therefore timing it with the onset of puberty. However, puberty is the first step in a series of processes leading to achieve successful sexual reproduction, and puberty *per se* is weakly associated with successful mating, pregnancy, or maternal care (Mirskaia and Crew, 1930; Chehab et al., 1997; Brust et al., 2015). Therefore, it seems more reasonable to match sexual maturity with the ability to reproduce and raise offspring successfully, that is achieved at the end of adolescence and marks the beginning of adulthood (Vidal, 2017). This transition is characterized not only by the maturation of the reproductive system, but also by changes in the endocrine balance and maturation of brain areas responsible for adult behavior involving social hierarchy, aggression, sexual repertoires, or offspring care (Sisk and Foster, 2004; Brust et al., 2015; Bell, 2018). The idea that adulthood starts after adolescence seems natural when we consider our own species, and probably the confusion around the onset of adulthood in murine rodents is related to the traditional disregard for the existence and extent of adolescence in those species (McCutcheon and Marinelli, 2009; Jackson et al., 2017). Indeed, abundant literature on the postnatal development of rodents in the last 3 decades shows that, like other mammals, they exhibit an adolescence period of transition between childhood and adulthood that paraphrasing Altman could be defined as transforming exuberant and reckless juveniles into cautious and observant adults (Altman et al., 1973; Spear, 2000, 2013; Adriani and Laviola, 2004; Caballero et al., 2016; Vanderschuren et al., 2016; Vidal, 2017; Bell, 2018; Laffan et al., 2018; Bara et al., 2021; Ghasemi et al., 2021). Adolescence in mice and rats starts shortly after weaning, with a rise in the levels of circulating gonadal hormones (Brust et al., 2015). These hormonal changes drive the onset of puberty, a hallmark of the adolescence period that marks the beginning of sexual reproduction. A subsequent period of post-puberal adolescence is characterized by critical hormonal and neurological changes that drive important physiological and behavioral changes (Romeo et al., 2002; Sisk and Foster, 2004; Spear, 2004; Vetter-O’Hagen and Spear, 2012; Bara et al., 2021) conductive to adult behavior and successful reproduction that mark the transition to adulthood. Still, young adults may not exhibit fully mature reproductive features [e.g., regular estrous cycles in female mice (Nelson et al., 1982)] that might take a few weeks to establish, marking the beginning of a peak reproductive period that might last several months. Subsequently, middle age can be characterized by the first signs of reproductive senescence (Mobbs et al., 1984; Silver, 1995; Tarín et al., 2014; Koebele and Bimonte-Nelson, 2016), starting with an initial phase of estrous irregularity, followed by persistent estrus, repeated pseudopregnancy (or persistent diestrus) and/or eventually, anestrus late in life (Vom Saal et al., 1994). The anestrous stage corresponds to a post-reproductive period that can last several months. Such long post-reproductive period is exceptional among mammals (Ellis et al., 2018), and might be largely influenced by favorable, stable environmental conditions and lack of predators.

## When are mice and rats adults?

**Figure 2:**
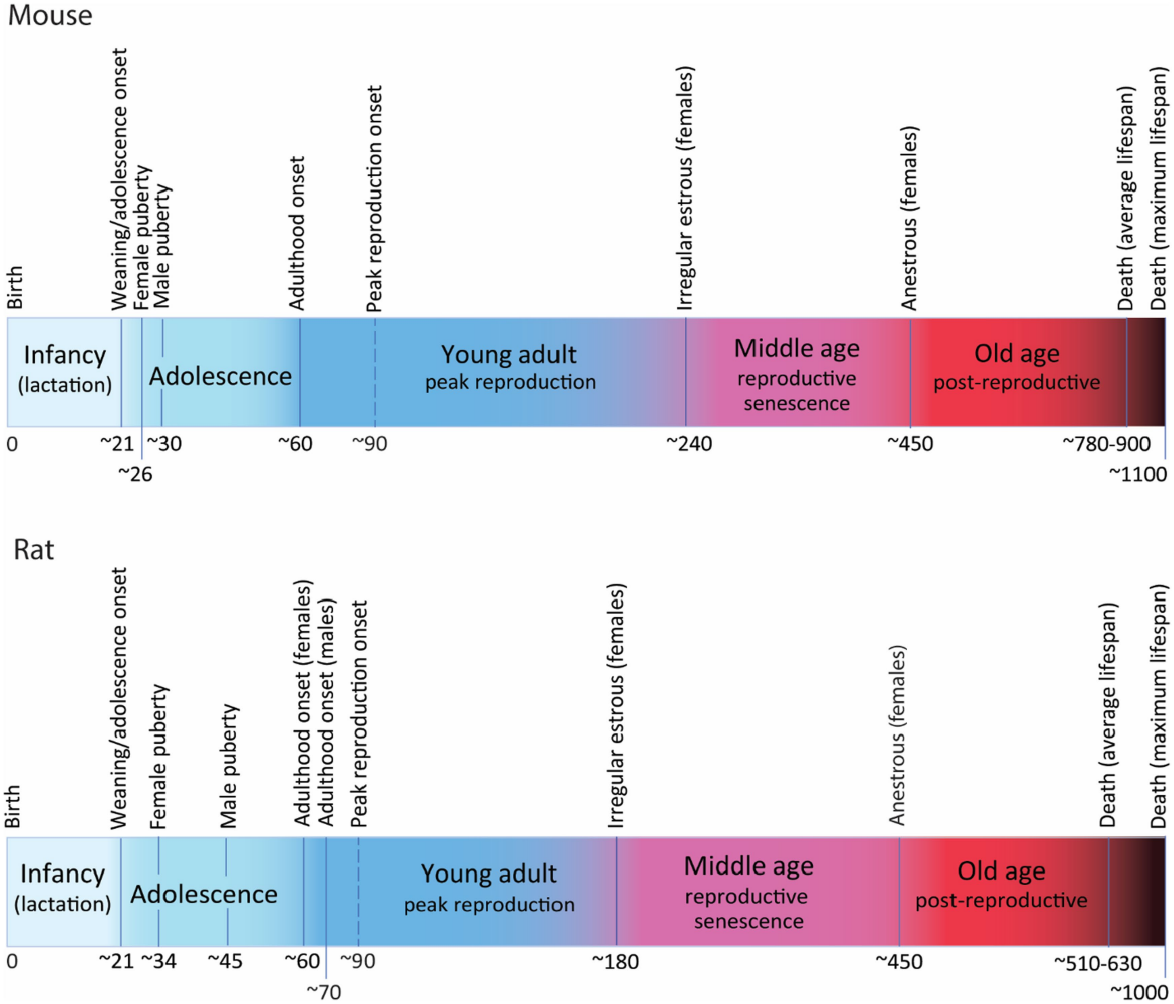
Postnatal developmental trajectory (not at scale) showing the most relevant milestones and periods along the lifetime of mice (C57) and rats (Wistar and Long Evans). Time in days. For detailed explanation see text.

Detailed research on the literature delving into the postnatal development of mice and rats shows interspecies differences and interstrain and interindividual variations within species. But beyond those differences, some guidelines can be reached regarding a timeline of developmental periods for both species:

Mice are weaned around P21, and soon after, between P21 and P23 mice enter adolescence (Brust et al., 2015; Figure 2). The onset of puberty is defined by vaginal opening in females and balano-preputial separation in males and is reached around P26 in females and about P30 in males (Safranski et al., 1993; Chehab et al., 1997; Brust et al., 2015; Sams et al., 2022), although sexual reproduction might be only achieved a few days later, after maturation of the genitalia and gametes (Safranski et al., 1993; Brust et al., 2015; Vidal, 2017). Post-puberal adolescence will encompass physiological, neurological and behavioral changes leading to sexual maturity, i.e., intratesticular testosterone in males reaches adult levels (Keene et al., 2002), and females are sexually and behaviorally mature to mate and raise offspring successfully (Brust et al., 2015). Post-puberal adolescence ends around P60, when mice reach adulthood (Laviola et al., 2003; Adriani and Laviola, 2004; McCutcheon and Marinelli, 2009; Brust et al., 2015). Peak reproduction is only achieved at age 3 months and lasts approximately until age 7–8 months (Finch, 2014). Reproductive senescence manifest around 8 months of age in C57 females with irregular estrous cycles that will evolve through subsequent phases to reach permanent anestrous marking the end of reproduction around age 13–15 months (Nelson et al., 1982; Gosden et al., 1983; Mobbs et al., 1984; Silver, 1995; Tarín et al., 2014; Koebele and Bimonte-Nelson, 2016). Average lifespan of mice also depends on strains but C57BL/6 mice, the most common inbred strain used in functional studies of AHN has an average lifespan of 26–30 months with maximum lifespan beyond 36 months (Kunstyr and Leuenberger, 1975; Jucker and Ingram, 1997; Yuan et al., 2011).

Rats have a similar trajectory, also weaning around P21 and entering adolescence soon after (Figure 2). Puberty onset is detected around P34 (P30–P39) in females and about P45 (P38–P48) in males (Rivest, 1991; Lewis et al., 2002; Ojeda and Skinner, 2006; Ghasemi et al., 2021) although there are reports describing large variation in puberty onset in males, from P40 to P76 (Lewis et al., 2002). As in mice, sexual reproduction might not be achieved until a few days later when gonads and gametes are more mature. Post-puberal adolescence extends until the onset of adulthood that has been timed around P60 (P56–P65) in females and P70 in males (Vetter-O’Hagen and Spear, 2012; Campion et al., 2013; Spear, 2013; Vidal, 2017; Laffan et al., 2018; Ghasemi et al., 2021). Reproductive senescence starts earlier in rats than in mice, with some reports indicating that irregular estrous can be detected as early as 6–7 months in some individuals (Everett, 1980; Nelson et al., 1982; Vom Saal et al., 1994; Shirai et al., 2015), that will evolve through the phases of reproductive senescence to reach anestrous by 15 months of age, although this timing is variable depending on strains (Vom Saal et al., 1994; Finch, 2014). Average lifespan of rats is also strain-dependent: about 17 months for Wistar rats (Langley-Evans and Sculley, 2006) and 21 months for Sprague Dawley (Xu et al., 2013), with documented maximum lifespan of 34 months in Wistar (Goodrick, 1980) and 29 months in Sprague–Dawley rats (Xu et al., 2013), the most commonly used strains.

## Discussion

Defining adulthood in any species might be difficult, as cessation of development and maturation are heterochronous events in different systems, organs and even in different structures within an organ, as described for the brain. However, it is necessary to define a conventional timepoint to reflect the onset of adulthood in a species, as an operational starting point for any meaningful experimental or theoretical approach. This is a particularly pressing issue in the field of AHN that requires a reliable definition of adulthood but has been characterized by lack of consistency. Our review of the available developmental data for mice and rats shows that beyond inter-strain variability, a generic timeline can be established that sets the onset of puberty around 1 month of age for females and slightly later for males, followed, like in other mammalian species, by a postpuberal adolescence period that ends around postnatal day 60 in both species, marking the onset of adulthood (McCutcheon and Marinelli, 2009; Brust et al., 2015). Therefore, based on reproductive and behavioral maturation, P60 seems to be the very minimum standard to mark the onset of adulthood in both mice and rats.

The data presented here are focused on reproductive physiology, but similar timing for the onset of adulthood has been reported when analyzing neurogenic features such as the ratio between dying and proliferating cells in the dentate gyrus (Amrein et al., 2011). However, it can be argued that this threshold does not fully represent the onset of adulthood in the sense of cessation of development and structural and functional stabilization, since rapid maturational growth in many biologic processes and structures continues beyond 2 months of age (Flurkey et al., 2007; Jackson et al., 2017). For example, estrous stabilization in mice is only achieved between 4 and 5 months (Nelson et al., 1982). Body mass and bone length and density in C57 mice has been shown to stabilize only between 4 and 6 months (Somerville et al., 2004; Fu et al., 2013); in the immune system, T and B-lymphocyte production increases until around 6 months of age (Kincade, 1981); in the nervous system, brain volume increases up to 3 months of age (Mandell et al., 2010); neocortical mass and neuronal number is stable during postnatal development, but the non-neuronal component stabilizes at 3 months of age and hippocampal mass plateaus at 3–4 months of age in mice (Fu et al., 2013); spinogenesis and synaptogenesis in the rat visual cortex experiences changes in density, number and length especially in symmetric synapses between 1 and 3 months of age (Miller, 1981; Blue and Parnavelas, 1983; Bähr and Wolff, 1985) and dopamine receptor expression reaches a maximum at 2 months and then decreases to stable levels, between 3 and 4 months, in the prefrontal cortex (McCutcheon and Marinelli, 2009). This heterochrony in the stabilization of different growth processes emphasizes the notion that maturation of the body and nervous system is a protracted process and not an event occurring at a specific age.

Nonetheless, for practical reasons, it is necessary to establish an age that reasonably reflects the transition to adulthood. Following reproductive and behavioral criteria this can be defined to be around 2 months of age for both rats and mice. Nevertheless, considering the ongoing developmental processes still active at age 2 months, and the maturational differences related to inter-sexual, interindividual, inter-strain and environmental and social factors, it seems reasonable to follow the proposal by researchers at The Jackson Laboratory (Flurkey et al., 2007) defining the onset of adulthood at 3 months of age, to warrant that any animal studied is a *bona fide* adult.

## Data availability statement

The original contributions presented in the study are included in the article/supplementary material, further inquiries can be directed to the corresponding authors.

## Author contributions

JA: Writing – original draft, Formal analysis, Data curation, Conceptualization. AD: Writing – review & editing. PR: Writing – review & editing, Resources, Funding acquisition.
